# Autophagy in PE: Dispute, Role and Potential Target

**DOI:** 10.1111/cpr.70102

**Published:** 2025-08-12

**Authors:** Miao Xu, Qi Wang, Fang Wang, Li Kang, Huijing Ma, Mengnan Li, Zhuanghui Hao, Zhengrui Li, Ji'an Liu, Xufeng Huang, Hengrui Liu, Shouxin Wei, Hailan Yang

**Affiliations:** ^1^ Department of Obstetrics The First Hospital of Shanxi Medical University Taiyuan China; ^2^ Department of Oncology Ruijin Hospital, Shanghai Jiao Tong University School of Medicine Shanghai China; ^3^ Department of Data Visualization, Faculty of Informatics University of Debrecen Debrecen Hungary; ^4^ Department of Biochemistry University of Cambridge Cambridge UK; ^5^ Department of Gastrointestinal Surgery Suining Central Hospital Suining China

**Keywords:** autophagy, immune response, inflammation, PE, placental development, trophoblast invasion, vascular remodelling

## Abstract

PE is a life‐threatening pregnancy disorder that can lead to adverse events for both the fetus and the mother. Autophagy is a cellular process involved in cellular renovation and maintaining homeostasis. There is a growing body of evidence suggesting that autophagy in trophoblasts plays a significant role in the development and pathogenesis of PE. However, the exact mechanisms are not yet fully understood. This article provides an overview of recent evidence regarding the role of autophagy in trophoblast invasion, vascular remodelling, inflammation, immune response, and maternal factors in the context of PE. It is believed that impaired or excessive autophagy can contribute to placental ischaemia and hypoxia, thereby exacerbating PE progression. Therefore, understanding the molecular mechanisms that regulate autophagy in PE is crucial for the development of targeted therapeutic interventions in the future.

## Graphical abstract

Autophagy homeostasis serves as a central hub regulating vascular remodelling during pregnancy. It integrates maternal, genetic, and fetal factors; shapes the functions of immune cells (NK cells, macrophages, and T cells); restrains endothelial inflammatory responses (cytokines, endothelial cell, oxidative/ER stress, and protein aggregation); and supports trophoblast invasion and survival—especially interstitial and endovascular extravillous trophoblast (EVT). Together, these processes promote spiral artery and overall vascular remodelling. Disruption of autophagy perturbs these interconnected pathways and may lead to pregnancy‐related vascular complications.

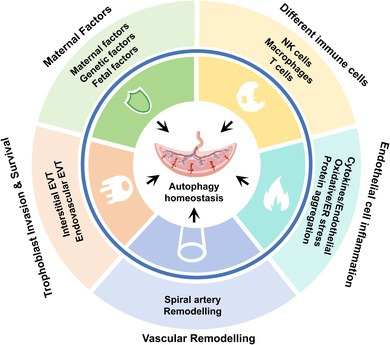

## 1 Introduction

Preeclampsia (PE) is a pregnancy‐specific disorder characterised by new‐onset hypertension and proteinuria after 20 weeks of gestation, often accompanied by signs of maternal organ dysfunction, such as renal impairment or pulmonary edema [1, 2]. Affecting approximately 2%–5% of pregnancies worldwide, it is a leading cause of maternal and perinatal morbidity and mortality, contributing to an estimated 46,000 maternal deaths and 500,000 fetal and newborn deaths annually [3, 4]. Women with a history of PE face an elevated risk of future cardiovascular diseases, while their offspring may experience higher rates of metabolic and cardiovascular disorders later in life [3, 4].

Given its significant impact on maternal and fetal health, effective management of PE is critical. Delivery of the baby and placenta remains the definitive intervention, as the condition typically resolves post‐delivery [5, 6]. However, clinical management strategies are tailored to the severity of the condition. For mild PE, expectant management with regular monitoring of maternal and fetal health is common, whereas severe PE necessitates intensive monitoring, antihypertensive medications, and often earlier delivery to prevent complications. The complexity of these strategies underscores the need to elucidate the pathological mechanisms of PE to enhance prenatal care and optimise outcomes [5, 6].

PE is recognised as one of the “great obstetrical syndromes,” characterised by multiple pathological processes converging on common pathways, including endothelial dysfunction, inflammation, and placental stress [7, 8]. Although the underlying pathophysiology might be quite different, early‐ and late‐onset PE as using different pathways to arrive at the final common endpoint, syncytiotrophoblast stress [9]. Autophagy plays a complex and multifaceted role in PE, where excessive autophagy induced by oxidative stress impairs trophoblast invasion and placental vasculature, while impaired autophagy exacerbates inflammation and reduces trophoblast resilience to oxidative stress, highlighting its critical involvement in the disease's pathogenesis [10, 11, 12].

Specifically, autophagy regulates trophoblast invasion, a critical process in placental development. Studies indicate that inhibiting autophagy can enhance trophoblast invasion, suggesting that excessive autophagy may contribute to the impaired invasion observed in PE [12, 13]. Additionally, autophagy modulates inflammation via the NF‐κB pathway; inhibiting autophagy increases NF‐κB activity, thereby affecting the production of inflammatory and angiogenic factors (e.g., sFlt‐1) [13]. In endothelial cells, oxidative stress‐induced excessive autophagy disrupts placental vascular formation, further linking autophagy to endothelial dysfunction in PE [14]. Thus, autophagy dysregulation is intricately associated with the key pathological mechanisms of endothelial activation, inflammation, and syncytiotrophoblast stress in PE.

While the association between autophagy and PE is increasingly recognised, the specific regulatory mechanisms of autophagy in the pathogenesis of PE are still being actively investigated. Recent studies imply that autophagy may be associated with PE through regulating trophoblast invasion [12], vascular remodelling [12], inflammation [15], immune response [11] and maternal factors [16] at the maternal‐fetal interface. This review highlights the importance of autophagy during the development of PE and provides new insights into PE and potential treatments.

## 2 Autophagy in Normal Pregnancy

To understand autophagy's role in pregnancy, a brief overview of its cellular function is necessary. Autophagy is an intracellular catabolic process that degrades molecules, damaged organelles, and intracellular pathogens [17]. There are three primary forms of autophagy: macroautophagy, microautophagy, and chaperone‐mediated autophagy (CMA). In this review, we refer to macroautophagy as autophagy, as it is the most extensively studied form in the context of PE, with substantial evidence linking it to placental dysfunction and disease mechanisms [17, 18, 19]. In contrast, the roles of microautophagy and CMA in PE remain largely unexplored, with no direct evidence currently implicating them in the disease's pathophysiology. This focus on macroautophagy reflects the predominance of research on this pathway, though future studies may elucidate potential contributions of microautophagy and CMA [20].

Autophagy orchestrates critical cellular processes during embryogenesis, implantation, placentation, and normal pregnancy maintenance [21]. In healthy pregnancy, autophagy continues to be expressed in human placentas throughout gestation [22]. LC3 and Beclin‐1 are located mainly in villous cytotrophoblast in early gestation, while at term they are found in villous syncytiotrophoblast [23, 24, 25, 26]. Additionally, autophagy activation is enhanced during syncytialisation, when cytotrophoblasts differentiate to form multinucleated mature syncytiotrophoblast [27, 28]. Autophagy can also protect the syncytiotrophoblast from bacterial infection [29], which is essential for placental homeostasis.

Autophagy can be summarised in four main steps: initiation, elongation, maturation, and degradation [18]. When autophagy is induced, the selected cargo or organelles are enclosed by a phagophore, followed by the formation of autolysosomes. Several autophagy‐related proteins (ATGs) orchestrate autolysosome formation. ATG4 mediates the conversion of LC3‐I (microtubule‐associated protein 1 light chain 3) to LC3‐II, and then LC3‐II anchors to the autolysosome for membrane elongation. Cargos are recognised by LIR‐containing selective autophagy adaptor SQSTM1, also known as p62, and are enclosed by autolysosomes. Then, membrane‐bound LC3 directs the growth, closure, and maturation of the autolysosome. ATG7 mediates ATG5‐ATG12‐ATG16L1 complex formation to facilitate autolysosome maturation [19]. Finally, autolysosomes fuse with lysosomes to form autolysosomes, in which the autolysosome contents are degraded and recycled for metabolic homeostasis [20].

## 3 Autophagy Dysregulation in PE

Accumulating evidence suggests that autophagy is dysregulated in PE, with studies reporting both increased and decreased autophagy activity in placental tissues from PE patients compared to normotensive pregnancies (Table 1). This dysregulation is thought to contribute to the pathological features of PE, such as impaired trophoblast invasion, endothelial dysfunction, and inflammation [11, 12, 15, 16].

**TABLE 1:** Dysregulated autophagy and alterations of related molecules in PE.

Study	Autophagy status	Human samples	Key molecular alterations	Affected pathways/mechanisms
Garcia‐Puente, 2024 [30]	↑	Placenta villous in LOPE	↑ ATG9A, LC3, LAMP‐1; ↓ p62	PI3K/Akt/mTOR (inhibits trophoblast invasion)
Luo, 2023 [31]	↑	Placenta villous in PE	↑ LC3‐II, Beclin1; ↓ p62	AMPK (promotes oxidative stress response)
Chu, 2023 [32]	↑	Placenta villous in PE	↑ Beclin1, ATG5, LC3‐II, autophagic vacuoles; ↓ p62	HIF‐1α (impairs iEVT invasion)
Öcal, 2023 [33]	↑	Placenta villous in PE	↑ Beclin1	Wnt/β‐catenin (disrupts trophoblast migration)
Tsai, 2021 [34]	↑	Placenta villous in PE	↓ mTOR; ↑ AMPK	AMPK/mTOR (enhances oxidative stress)
Ermini, 2021 [35]	↑	Placenta villous in EOPE	↑ Lysosomes, Beclin1, ATG9b, LAMP1, TFEB, L‐SMPD1	TFEB‐mediated lysosomal biogenesis (impairs enEVT vascular remodelling)
Ausman, 2018 [36]	↑	Placenta villous in PE	↑ PINK1, CERs	PINK1‐mediated mitophagy (increases oxidative stress)
Pan, 2018 [37]	↑	Placenta villous in severe PE	↑ LC3‐II; ↓ p62	PI3K/Akt/mTOR (inhibits iEVT invasion)
Xu, 2017 [38]	↑	Placenta villous in severe PE	↑ LC3‐II/I, Atg4B	Autophagy‐lysosome pathway (impairs trophoblast survival)
Akcora Yildiz, 2017 [39]	↑	Placenta villous in PE	↑ Beclin1, p62	NF‐κB (promotes inflammation)
Hutabarat, 2017 [40]	↑	Placenta villous in EOPE	↑ LC3B, Beclin‐1	HIF‐1α (impairs iEVT invasion)
Prokesch, 2017 [41]	↑	Placenta villous in EOPE	↑ LC3B‐I, LC3B‐II	Autophagy‐lysosome pathway (disrupts enEVT remodelling)
Gao, 2015 [14]	↑	Placenta villous in PE	↑ LC3, Beclin1, autolysosome	AMPK (enhances oxidative stress)
Akaishi, 2014 [42]	↑	Placenta villous in PE	↑ LC3‐II; ↓ p62	PI3K/Akt/mTOR (impairs iEVT invasion)
Kalkat, 2013 [43]	↑	Placenta villous in severe EOPE	↑ LC3‐II, Beclin‐1, autolysosomes	HIF‐1α (disrupts trophoblast invasion)
Oh, 2008 [44]	↑	Placenta villous in severe PE	↑ LC3‐II; Beclin‐1 unchanged	mTOR (impairs trophoblast survival)
Sun, 2023 [45]	↓	Placenta villous in PE	↓ PINK1, LC3B; ↑ p62	PINK1‐mediated mitophagy (increases oxidative stress)
Weel, 2023 [46]	↓	Placenta villous in PE	↓ LC3‐II, Beclin‐1; ↑ mTOR	mTOR (impairs iEVT invasion)
Ribeiro, 2023 [47]	↓	Placenta villous in PE	↑ p62, NF‐κB	NF‐κB (promotes inflammation)
Cheng, 2022 [48]	↓	Placenta villous in severe EOPE	↑ p62, ubiquitinated proteins, TTR	Autophagy‐lysosome pathway (impairs trophoblast survival)
Zhou, 2021 [49]	↓	Placenta villous in PE	↓ BNIP3, mitophagy; ↑ LC3‐II/I, p62, cathepsin D	BNIP3‐mediated mitophagy (increases oxidative stress)
Vangrieken, 2021 [50]	↓	Placenta villous in PE	↑ p62, BNIP3; ↓ PGC‐1α; no change in PINK1, FUNDC1	Mitochondrial dysfunction (impairs enEVT remodelling)
Li, 2021 [51]	↓	Placenta villous in EOPE	↓ LC3B, Beclin1; ↓ autolysosomes	Autophagy‐lysosome pathway (impairs trophoblast survival)
Nakashima, 2020 [52]	↓	Placenta villous in PE	↑ p62; ↓ LAMP1/2, cathepsin D, nuclear TFEB	TFEB‐mediated lysosomal biogenesis (impairs iEVT invasion)
Ozsoy, 2018 [53]	↓	Placenta villous in PE	↓ p97/VCP; ↑ LC3‐II, p62, ubiquitinated proteins	Autophagy‐lysosome pathway (impairs trophoblast survival)
Nakashima, 2013 [54]	↓	Placenta villous in PE	↑ p62	Autophagy‐lysosome pathway (impairs iEVT invasion)

Abbreviations: Akt, protein kinase B, also known as AKT; AMPK, adenosine 5′ monophosphate‐activated protein kinase; ATG, autophagy‐related proteins; BNIP3, BCL2/adenovirus E1B19kDa protein‐interacting protein 3; enEVT, endovascular extravillous trophoblast cell; EOPE, early‐onset PE; FUNDC1, FUND14 domain containing 1; HIF‐1α, Hypoxia‐inducible factor 1 α; iEVT, interstitial extravillous trophoblast cell; LAMP, lysosomal‐associated membrane protein; LC3, Microtubule‐associated protein 1 light chain 3; LOPE, late‐onset PE; mTOR, mammalian target of rapamycin; NF‐κB, Nuclear factor kappa‐B; PE, PE; PGC‐1, peroxisome proliferator‐activated receptor γ coactivator 1; PI3K, Class III phosphatidylinositol 3‐kinase complex; PINK1, PTEN induced putative kinase 1; TFEB, transcription factor EB.

In PE placentas, various autophagy‐related proteins have been found to be aberrantly expressed. For instance, increased levels of LC3‐II, Beclin1, ATG9, ATG5, LAMP1, and TFEB have been observed, indicating enhanced autophagy activity [30, 31, 32, 33, 34, 35, 36, 37, 38, 39, 40, 41, 42, 43, 44]. Ultrastructural analyses have also revealed a higher number of autophagic vacuoles in the syncytiotrophoblast layer of PE placentas [14, 32, 35, 43]. Conversely, some studies have reported decreased expression of LC3 and Beclin1, or increased p62 levels, suggesting impaired autophagy flux [45, 46, 47, 48, 49, 50, 51, 52, 53, 54]. These conflicting findings may reflect differences in the stage or severity of PE, as well as variations in methodological approaches.

### 3.1 Temporal Regulation of Autophagy Across Pregnancy Stages

Autophagy exhibits stage‐specific roles during pregnancy and the progression of PE. In early pregnancy (first trimester), autophagy is essential for supporting trophoblast invasion and vascular remodelling under hypoxic conditions, as evidenced by studies showing autophagy activation in extravillous trophoblasts around week 7 of gestation [55]. Dysregulation at this stage can lead to shallow trophoblast invasion, a hallmark of PE. As pregnancy progresses to the second and third trimesters, the role of autophagy shifts toward maintaining placental homeostasis by clearing damaged organelles and mitigating oxidative stress. However, in PE, this dynamic balance is disrupted. Recent studies suggest that in early‐onset PE (diagnosed before 34 weeks of gestation), autophagy is often suppressed, contributing to placental insufficiency by impairing the clearance of damaged cellular components, as evidenced by decreased LC3‐II and increased p62 levels [48, 51]. In contrast, in late‐onset PE (diagnosed after 34 weeks), excessive autophagy may occur, potentially leading to cellular damage and exacerbating systemic symptoms, with some studies reporting increased autophagy markers such as LC3‐II and Beclin1 [30]. For example, a lower LC3B/Beclin‐1 ratio has been observed in early‐onset PE compared to late‐onset PE, suggesting impaired autophagy flux in the former. These stage‐specific differences highlight the need for tailored therapeutic approaches based on the timing of disease onset [30, 48, 51].

### 3.2 Differential Regulation in PE Subtypes (Early‐Onset vs. Late‐Onset)

Early‐onset and late‐onset PE are distinct subtypes with differing aetiologies and placental pathologies. Recent research has begun to elucidate how autophagy, a critical cellular process for maintaining homeostasis, is differentially regulated in these conditions [55].

Early‐onset PE, typically diagnosed before 34 weeks of gestation, is often associated with severe placental dysfunction and fetal growth restriction. Studies consistently report impaired autophagy in the placentas of women with early‐onset PE. Research demonstrates a significant reduction in autophagosomes and autolysosomes, accompanied by increased protein aggregation and apoptosis [56]. This impairment is likely driven by chronic hypoxia and oxidative stress, which overwhelm autophagic capacity early in pregnancy. Molecular analyses reveal accumulation of p62, a marker of disrupted autophagy flux, alongside decreased expression of autophagy‐related proteins such as Atg12‐Atg5, Atg16L1, Atg3, Atg101, and GABARAP [54]. These findings suggest that defective autophagy contributes to poor placentation, including shallow trophoblast invasion.

In contrast, late‐onset PE, which typically manifests after 34 weeks and is more closely linked to maternal factors such as endothelial dysfunction, exhibits a complex pattern of autophagy regulation. Recent studies have identified increased expression of autophagy initiation markers, including ULK1, ATG9A, LC3, ATG5, STX‐17, and LAMP‐1, in placentas from late‐onset PE cases [30]. These findings suggest that autophagy may be upregulated as a compensatory response to cellular stress. However, the overall autophagy flux remains controversial. Some investigations report elevated p62 levels, indicating potential impairment in autophagic degradation, while others observe increased MAP1LC3‐II and reduced p62, suggestive of active autophagy [55]. Additionally, suppressed expression of BNIP3, a key regulator of mitophagy, has been noted, leading to the accumulation of damaged mitochondria and increased apoptosis [49, 50]. These discrepancies may arise from variations in study methodologies or sample characteristics, highlighting the need for further research to clarify the autophagic status in late‐onset PE.

The differential regulation of autophagy in early‐ and late‐onset PE highlights their distinct pathophysiological mechanisms. In early‐onset PE, impaired autophagy likely contributes to placental dysfunction, suggesting that therapies aimed at restoring autophagic function could be beneficial. In late‐onset PE, the upregulation of autophagy initiation markers may reflect a compensatory mechanism, but the potential impairment of autophagy flux indicates that simply enhancing autophagy may not be sufficient. The role of mitophagy, particularly in late‐onset PE, also warrants further exploration, as mitochondrial dysfunction appears to play a significant role.

### 3.3 Spatial Regulation Across Placental Regions (Fetal vs. Maternal Side)

The placenta is a heterogeneous organ with distinct functional zones, and autophagy regulation varies between the fetal side (e.g., syncytiotrophoblast and cytotrophoblast layers) and the maternal side (e.g., decidual cells and maternal‐fetal interface). Multiple studies have observed significantly elevated autophagy activity in preeclamptic placentas compared to normal pregnancies, indicating activation of autophagy [14, 30, 31, 32, 33, 34, 35, 36, 37, 38, 39, 40, 41, 42, 43, 44]. Immunocytochemical studies have localised several autophagy‐related gene products (LC3, Beclin1, ATG9, ATG5, LAMP1, TFEB) predominantly in the syncytiotrophoblast layer on the fetal side of preeclamptic placentas, where they are often upregulated [30, 33, 37, 40, 41, 44, 47]. Transmission electron micrographs have also revealed increased numbers of autophagic vacuoles accompanied by lysosomes in these layers [14, 32, 35, 43]. This suggests that autophagy may be hyperactivated on the fetal side as a response to stress induced by impaired placentation. In contrast, fewer studies have focused on the maternal side, but emerging evidence indicates that autophagy may be suppressed in decidual cells at the maternal‐fetal interface in PE, potentially contributing to defective spiral artery remodelling [54]. This spatial disparity highlights the importance of considering regional differences within the placenta when studying autophagy in PE. Future research should aim to map autophagy activity across placental zones to better understand its localised impact on PE pathogenesis [57].

### 3.4 Factors Contributing to Autophagy Dysregulation in PE

Several factors have been implicated in the dysregulation of autophagy in PE, which may further interact with temporal and spatial variations:

*Endoplasmic Reticulum (ER) Stress*: Chronic ER stress, particularly in early‐onset PE, may overwhelm autophagic capacity, leading to cell death. ER stress markers are upregulated in PE, disrupting lysosomal homeostasis and blocking autophagic flux in trophoblast cells [15, 48]. Moreover, the severity of ER stress correlates with the severity of PE, as chronic ER stress is more pronounced in severe cases, contributing to higher maternal morbidity and fetal growth restriction.

*Protein Aggregation*: Impaired autophagy in PE may lead to the accumulation of misfolded proteins, exacerbating cellular stress [48, 52]. Deficiency in aggrephagy, a selective form of autophagy, contributes to protein aggregation in preeclamptic placentas, potentially leading to poor placentation [48, 52]. The extent of protein aggregation is more significant in severe PE cases, directly correlating with the degree of placental dysfunction.

*Cellular Senescence*: Premature senescence of trophoblasts in PE is associated with altered autophagy. Senescent cells often exhibit defective autophagy, which may contribute to placental dysfunction [58]. Early‐onset PE, which is typically more severe, shows a stronger association with cellular senescence and autophagy impairment.

*Inflammation*: Excessive inflammation can dysregulate autophagy, while impaired autophagy can exacerbate inflammatory responses [9]. The intensity of inflammation is closely related to the severity of PE, with more severe cases exhibiting heightened NF‐κB signalling and inflammatory responses.

*Age and BMI*: Maternal age over 35 and higher BMI are associated with overexpression of autophagy‐related genes such as PKM and LEP, indicating exacerbated autophagy dysfunction (Autophagy‐related biomarkers). These factors are linked to more severe PE, particularly in cases with metabolic stress and immune dysregulation.

Overall, the transition of autophagy from a protective to a detrimental role likely depends on the stage of pregnancy, the subtype of PE, and the specific placental region affected. Larger‐scale studies with well‐designed protocols are needed to elucidate the exact relationship between autophagy and PE, incorporating temporal, subtype‐specific, and spatial analyses to inform targeted therapeutic strategies.

## 4 Autophagy and Trophoblast Invasion and Survival

Trophoblast invasion is a crucial step in embryonic implantation and placenta development. During blastocyst implantation, trophoblasts differentiate into cytotrophoblasts (CTBs) and syncytiotrophoblast (STB). CTBs further differentiate into extravillous and villous trophoblasts (EVTs). Villous trophoblast cells cover the chorionic villi and control the exchange of gas and nutrients between the mother and fetus [58, 59, 60]. EVTs invade the uterine wall and spiral arteries, but also invade uterine veins and uterine lymph vessels, which ensures a continuous blood supply to the placenta throughout pregnancy [61]. Specifically, EVTs comprise interstitial EVTs (iEVTs), which invade the decidual stroma to anchor the placenta, and endovascular EVTs (enEVTs), which remodel maternal spiral arteries to enhance blood flow to the placenta [62, 63, 64]. The differentiation and function of iEVTs are induced by factors such as hypoxia (mediated by HIF‐1α), growth factors (e.g., TGF‐β, IGF‐II, EGF), and decidual signals (e.g., IL‐8, CCL14). For enEVTs, inducing factors include hypoxia, angiogenic factors (e.g., VEGF, PLGF, ANGPT2), and endothelial signals (e.g., Notch, VCAM‐1) [65, 66].

Research indicates that autophagy plays a critical role in trophoblast invasion and survival, and its dysregulation may contribute to poor placentation in PE. Autophagy not only supports trophoblast survival by maintaining cellular homeostasis but also supports the survival of trophoblasts in the low oxygen and nutrient environment of early pregnancy, ensuring proper placental development [12, 67]. Experimental evidence from human trophoblast cells underscores this role. For instance, Saito et al. demonstrated that in primary cultured EVTs from human term placentas, autophagy is significantly enhanced under 2% oxygen conditions, mimicking early pregnancy hypoxia [12]. Autophagy‐deficient EVTs exhibited reduced invasion and vascular remodelling under these conditions, and this defect could be partially rescued by ATP treatment, highlighting the importance of autophagy in providing energy for trophoblast functions [12, 68]. These human sample studies are complemented by animal models, such as the placenta‐specific Atg7 conditional knockout mice generated by Aoki et al., which showed reduced trophoblast invasion and abnormal spiral artery remodelling due to impaired autophagy [67]. Nakashima et al. constructed autophagy‐deficient HTR8/HchEpC1b ‐ATG4^BC74A^ EVT cell lines to simulate EVT invasion in vitro, revealing that autophagy inhibition reduces MMP‐2 and MMP‐9 activity, impairing matrix degradation and invasion in iEVT‐like cells [54]. In contrast, enEVT‐like cells from first‐trimester placental explants suggest that autophagy supports spiral artery remodelling by promoting cell survival under hypoxic conditions, though its regulation of angiogenic factors like VEGFA remains under investigation.

**FIGURE 1:**
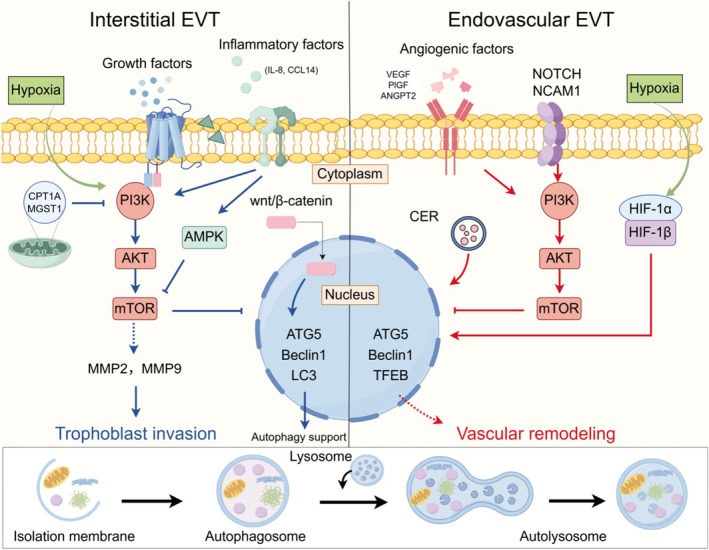
The autophagy pathways regulate trophoblast cell invasion and vascular remodelling. This figure illustrates the molecular mechanisms regulating trophoblast cell (EVT) functions, divided into interstitial EVTs (iEVTs, left) and endovascular EVTs (enEVTs; right), with a focus on autophagy support. iEVTs, which invade the decidual stroma to anchor the placenta, are induced by hypoxia (via HIF‐1α), growth factors (e.g., TGF‐β, IGF‐II, EGF), and decidual signals (e.g., IL‐8, CCL14), activating PI3K/AKT/mTOR and wnt/β‐catenin pathways to promote invasion through MMP2 and MMP9. enEVTs, which remodel maternal spiral arteries to enhance placental blood flow, are driven by hypoxia, vascular remodelling factors (e.g., VEGF, PLGF, ANGPT2), and endothelial signals (e.g., Notch, NCAM1), engaging PI3K/AKT/mTOR and HIF‐1α/HIF‐1β pathways for vascular remodelling. Autophagy, depicted by the formation of isolation membranes, autophagosomes, and autolysosomes, is supported by key proteins within the nucleus, facilitating both iEVT and enEVT functions.

At the molecular level, the process of trophoblast invasion is intricately governed by the crosstalk of multiple autophagy‐related signalling pathways, including PI3K/Akt/mTOR, AMPK, HIF‐1 and Wnt/β‐catenin (Figure 1). The PI3K/Akt/mTOR pathway, a central regulator of cell growth and metabolism, also controls autophagy; its activation typically inhibits autophagy, while its inhibition promotes autolysosome formation [69]. In trophoblasts, this pathway is modulated by transmembrane receptors such as ELABELA (ELA), ROR1, and DR3, which influence invasion in the context of PE [70, 71, 72]. Mitochondrial enzymes like microsomal glutathione S‐transferase 1 (MGST1) and carnitine palmitoyltransferase 1A (CPT1A) also activate this pathway to enhance invasion [73, 74]. In addition, STX2 and ANXA4 promote trophoblast invasion via the PI3K/Akt pathway in primary human trophoblast cells [75, 76]. Evidence shows that cytotrophoblasts isolated from PE placentas exhibit normalised gene expression, including SEMA3B, which is regulated through the PI3K/AKT and GSK3 pathways and contributes to trophoblast function [69]. These genes and receptors, such as ELA, ROR1, DR3, STX2, ANXA4, and SEMA3B, are primarily associated with trophoblast invasion, particularly in iEVTs [69, 70, 71, 72, 73, 74, 75, 76].

Beyond the PI3K/Akt/mTOR axis, other pathways also contribute to autophagy‐mediated trophoblast invasion. AMP‐activated protein kinase (AMPK), functioning as an energy sensor, promotes invasion by inhibiting mTOR and upregulating autophagic genes, thereby supporting trophoblast function under energy stress [77]. During the first trimester of pregnancy, moderately upregulated HIF‐1α serves as an energy source for EVT invasion via activating autophagy [78]. However, researchers have observed that overexpression of HIF1α decreases the invasiveness of autophagy‐deficient HTR8/SVneo cells through the suppression of MMP‐9, which can be recovered by ATP treatment [79]. Lastly, the Wnt/β‐catenin pathway further enhances invasion by upregulating MMPs, with potential intersections with autophagy regulation [80]. Emerging evidence suggests that Wnt/β‐catenin signalling may also contribute to enEVT differentiation and spiral artery remodelling, though its interaction with autophagy in this context requires further exploration [81, 82].

Recent studies emphasise the importance of distinguishing iEVTs and enEVTs due to their distinct roles in placental development. Autophagy is likely critical for both subtypes, supporting their survival and function under hypoxic conditions. In iEVTs, autophagy enhances matrix degradation and invasion, as evidenced by reduced MMP‐2 and MMP‐9 activity in autophagy‐deficient models [83]. Key autophagy genes such as ATG5, Beclin‐1, and LC3 are upregulated in iEVTs to maintain cellular homeostasis. In enEVTs, autophagy may support spiral artery remodelling by promoting cell survival and potentially interacting with transcription factors like EPAS1, TFAP2C, and SNAI1, which are implicated in EVT development and vascular remodelling [84]. Additionally, enEVTs express markers such as NCAM1 and HLA‐G, distinguishing them from iEVTs. However, the specific autophagy‐related genes (e.g., ATG5, Beclin‐1) in enEVTs are less characterised, and their regulation via pathways like Notch and HIF‐2α remains understudied. Future research, including single‐cell RNA sequencing, is needed to elucidate subtype‐specific mechanisms [63, 84].

In conclusion, trophoblast invasion is a multifaceted process critically dependent on autophagy, particularly in the low oxygen environment of early pregnancy. Dysregulation of autophagy—whether through deficiency or excess impairs trophoblast migration and invasion, contributing to placental pathologies such as PE. Experimental evidence from both human samples and animal models highlights the indispensable role of autophagy in maintaining trophoblast function and placental development. Understanding these mechanisms may provide insights into the pathogenesis of pregnancy complications and pave the way for novel therapeutic approaches.

## 5 Autophagy in Vascular Remodelling

Spiral artery remodelling, the physiological transformation of uterine spiral arteries into highly dilated thin‐walled vessels, is vital to human pregnancy development [59]. Vascular remodelling consists of five stages: endothelial vacuolation, invasion of stromal and perivascular tissues by interstitial trophoblast, endovascular trophoblast migration, incorporation of endovascular trophoblast into the vessel wall, and maternal vascular repai; reendothelialization occurs [59, 85]. Its unique feature is that the endothelium disappears progressively and is replaced by trophoblast and the deposition of a fibrofibrinoid structure, finally developing a 200–500 mm diameter uteroplacental spiral artery [86]. Brosens et al. confirmed that in normal pregnancy physiological changes extend from the decidual terminations of the spiral arteries as far as the radial arteries deep into the myometrium, except for the periphery of the placental bed [87, 88, 89]. They also documented that in the presence of PE, physiological changes are reduced and occur only in the central portion of the placental bed [87, 88]. Autophagy is essential for vascular remodelling and placental development in PE [12, 89].

Excessive autophagic activity has been associated with inadequate vascular remodelling in PE [89]. Autophagy is essential for the survival and function of vascular endothelial cells and smooth muscle cells, which are core components of the vascular system necessary for proper remodelling [21]. Lee et al. showed that impaired autophagic flux in the uterine vascular mural cells and fibroblasts in atg7^d/d^ mice may lead to the accumulation of vascular factors, including VEGFA and NOS1, wherein VEGFA‐KDR signalling and NO overproduction by NOS1 lead to junctional instability in autophagy‐intact endothelial cells [90]. Dysregulation of autophagy may lead to impaired vascular adaptation, contributing to the hypertensive phenotype observed in PE [67]. Gao et al. demonstrated that the reduction of HTR8/Svneo cells invasion and endothelial cell tube formation index was significantly reduced following treatment with glucose oxidase, which was partially reversed by 3‐MA (an inhibitor of autolysosome formation) in vitro [14]. In 2020, Zhao et al. induced a selective PKCβ‐inhibited PE‐like mice; excessive autophagic flux and an angiogenic imbalance were identified in mouse placentas, which could also be blocked by 3‐MA [91]. Their findings suggest that autophagy mediated, at least in part, the impairment of angiogenesis in PKCβ inhibition‐induced PE [91]. TFEB also plays a critical role in normal placenta vascularisation, as homozygous TFEB KO mice displayed defects in the ability of the embryonic vasculature to invade the placenta [92]. Ermini et al. demonstrate that TFEB‐induced lysosome biogenesis is markedly increased in the syncytiotrophoblast layer of early‐onset preeclamptic placentae, accompanied by augmented lysosomal exocytosis and ceramide accumulates [35]. Furthermore, they employed in vitro and in vivo experiments showing that ceramide increases TFEB expression and nuclear translocation and induces lysosomal formation and exocytosis [35]. In addition, ceramide‐induced lysosomal exocytosis carries L‐SMPD1 to the apical membrane of the syncytial epithelium, resulting in ceramide accumulation in lipid rafts and the release of active L‐SMPD1 via ceramide‐enriched exosomes into the maternal circulation. L‐SMPD1 in the released exosomes promotes endothelial activation and impairs vascular remodelling, thereby contributing to the endothelial dysfunction in early‐onset PE women [35].

However, impaired autophagy is possibly relevant to the failure of vasculature [89]. Nakashima et al. also showed that the failure of vascular remodelling in autophagy‐deficient cell lines under hypoxic conditions in vitro [54]. In 2018, Aoki et al. demonstrated that placentas with deficient autophagy‐related gene 7 (Atg7) exhibit failure of vascular remodelling, leading to placental growth restriction [67]. In 2021, Li et al. presented that autophagy activation by hypoxia could rescue impaired angiogenesis and apoptosis in ox‐LDL‐mediated PE in vitro EVT model [51]. Additionally, autophagy activation by hypoxia has been demonstrated to rescue impaired vascular remodelling by differentially regulating the expression of VEGFA and FLT1 [93]. The chemokine C‐X‐C motif ligand 12 (CXCL12)– chemokine C‐X‐C motif receptor 4 (CXCR4) axis is central to VEGFA signalling, and altering CXCL12–CXCR4 signalling at the fetal‐maternal interface results in locally diminished vascularization and induction of autophagy, which plays vital roles in proper placentation [93]. These findings collectively emphasise the protective role of autophagy in EVT function and vascular remodelling during placental development.

Hence, autophagy plays a dual role in placental development and vascular remodelling in PE. Autophagy changes and trophoblast injuries may occur in different subpopulations of trophoblast cells: villous trophoblast or extravillous trophoblast, which may contribute to the same pathological process. While excessive or impaired autophagy activity induces the failure of vasculature, there is possible relevance to the pathogenesis of PE.

## 6 Autophagy in Endothelial Cell Inflammation

### 6.1 Autophagy Regulates Inflammatory Cytokines Response

**FIGURE 2:**
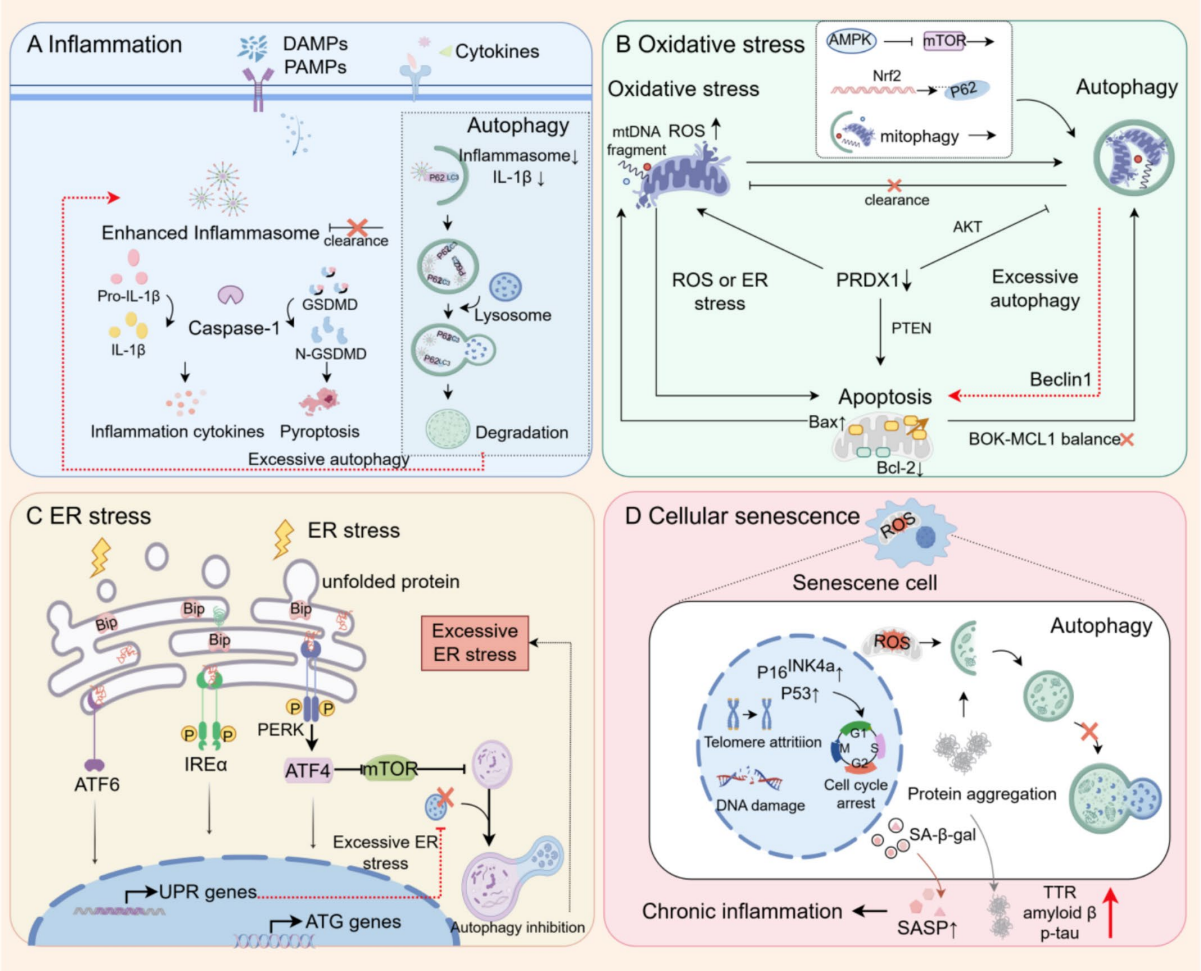
Interaction between autophagy and inflammation. The elevation of autophagy in early PE might initially stem from an adaptive response to intracellular stress or injury. Nonetheless, excessive autophagic activity could itself inflict damage on trophoblasts, leading to the excessive and systemic inflammatory reactions, including cytokines, oxidative stress, ER stress and protein aggregation in PE.

Cytokines, secreted by both innate and adaptive immune cells, serve not only as critical regulators of these cells' functions and survival but also drive critical pathophysiological processes, including inflammation and vascular dysfunction (Figure 2). Among these, pro‐inflammatory cytokines such as Interleukins (IL)‐1, IL‐2, IL‐6, Tumour Necrosis Factor (TNF)‐α, and Interferon (IFN)‐γ drive the pathogenesis of preeclampsia (PE) by promoting systemic inflammation and endothelial dysfunction [94, 95, 96, 97, 98]. Multiple studies have demonstrated significantly elevated levels of these pro‐inflammatory cytokines in the sera of PE patients, which coincide with clinical manifestations of endothelial dysfunction and molecularly highlight inflammation's central role in the disease's pathogenesis [7, 15, 99, 100, 101].

Recent research has further illuminated distinctive inflammatory features within PE‐placental tissue: besides the upregulation of the aforementioned cytokines, it has observed the activation of NLRP3 and AIM2 inflammasomes, along with pronounced increases in pyroptosis proteins [102]. These inflammasomes, functioning as pattern recognition receptors, recognise and respond to intracellular pathogens or damage‐associated molecular patterns [102]. They amplify inflammatory signals and promote cytokine release through the induction of pyroptosis, a form of programmed cell death, thereby fueling both local and systemic inflammatory responses [102].

Autophagy, an evolutionarily conserved cellular degradation pathway, primarily involves the encapsulation and breakdown of targeted substrates, including inflammatory mediators and signalling molecules [19]. In theory, augmented autophagy activity should attenuate inflammation by eliminating drivers such as the NLRP3 inflammasome, thereby decreasing the production of pro‐inflammatory agents like IL‐1β. However, in the early stages of PE, a paradoxical scenario arises in trophoblast cells: despite heightened autophagy levels, pro‐inflammatory cytokines do not correspondingly decline; instead, both autophagy and cytokine activation seem exaggerated [33, 41, 44].

To address this paradox, we propose the following hypothesis: the elevation of autophagy in early PE trophoblasts might initially stem from an adaptive response to intracellular stress or injury. Nonetheless, excessive autophagic activity could itself inflict damage on some trophoblast cells, leading to the release of damage‐associated molecular patterns that ignite a localised inflammatory reaction. This process attracts and activates infiltrating inflammatory cells, such as macrophages and natural killer cells, prompting them to abundantly produce cytokines like IL‐1β, TNF‐α, and IFN‐γ, which exert broad pro‐inflammatory effects. Importantly, these cytokines not only directly exacerbate inflammation but also stimulate the NLRP3 and AIM2 inflammasomes and their downstream pyroptotic pathways within trophoblasts, amplifying inflammatory signalling in placental tissue. Consequently, a vicious cycle ensues where inflammation and autophagy mutually drive each other.

This hypothesis offers explanations for several critical phenomena:

1. It explains the aberrantly high expression of pro‐inflammatory cytokines in the sera of PE patients, suggesting that these cytokines originate not only from immune cells but also from autophagy‐induced trophoblast injury.
2. It elucidates why cytokine levels in the placenta fail to decrease despite enhanced autophagy, revealing that cytokine generation and release are positively regulated by the autophagy‐induced inflammatory response.
3. It clarifies how increased placental cytokines, in turn, feedback to promote autophagy levels, reinforcing the reciprocal relationship between autophagy and inflammation and establishing a persistent, self‐perpetuating pathological state that is difficult to reverse.

Of course, further experimental validation in multiple settings is necessary to substantiate this hypothesis.

### 6.2 Autophagy in Endothelial Cell Inflammation in PE

**FIGURE 3:**
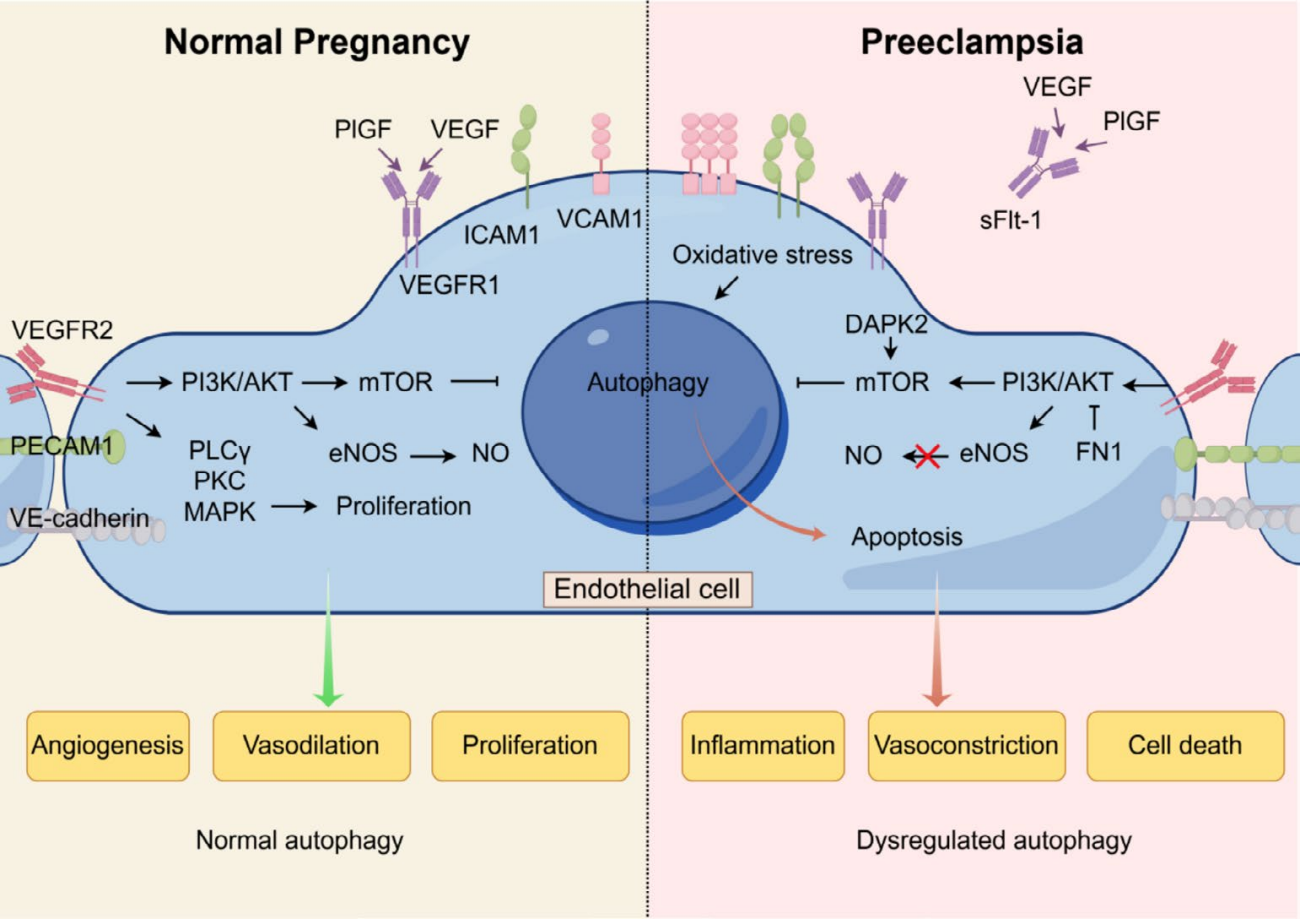
Endothelial function and dysfunction: The role of autophagy. Endothelial cell responses to normal pregnancy (Normal autophagy; left) and Preeclampsia (dysregulated autophagy; right). Normal autophagy induces endothelial homeostasis through the PI3K/Akt and MAPK signalling pathway. The PI3K/Akt pathway then increasing vasodilation as well as reduce inflammatory processes while increasing angiogenesis. The MAPK promotes endothelial cell proliferation. Dysregulated autophagy promotes an increase in inflammatory (increased endothelial markers ICAM1 and VCAM1), endothelial cell death (apoptosis) and vasoconstriction (reduces NO biosynthesis). Three pathways have been identified: (i) oxidative stress activates autophagy‐related proteins, which meanwhile reduced angiogenesis; (ii) DAPK2 enhances mTOR signalling, which lose the protection to apoptosis; and (iii) FN1 promoted apoptosis and autophagy by inhibiting the PI3K/AKT/mTOR signalling.

Endothelial dysfunction is a hallmark feature of PE, characterised by increased vascular permeability, activation of coagulation pathways, and inflammation [7, 8, 9]. Autophagy plays a critical role in maintaining endothelial cell homeostasis, and its dysregulation may contribute to endothelial dysfunction (Figure 3) [14]. Research suggests that in PE, oxidative stress induces increased autophagy in endothelial cells. For instance, Gao et al. demonstrated that treatment of human umbilical vein endothelial cells (HUVECs) with glucose oxidase (which generates oxidative stress) led to increased expression of autophagy‐related proteins LC3 and Beclin‐1, as well as an increased number of autolysosomes [14]. This excessive autophagy was associated with reduced endothelial cell tube formation, a key indicator of angiogenesis and vascular function. Similarly, in placentas from early‐onset preeclamptic pregnancies, LC3 and Beclin‐1 expression was significantly higher than in normal pregnancies [35, 40, 41]. Wang et al. reveal that DAPK2 enhances mTOR signalling to protect placental cells from oxidative damage and apoptosis by inhibiting autophagy in human placental microvascular endothelial cells [103]. In vitro experiments suggested that FN1 promoted apoptosis and autophagy in HUVECs by inhibiting the PI3K/AKT/mTOR signalling pathway, which may be the mechanism underlying its involvement in the pathogenesis of PE [104]. These findings suggest that oxidative stress‐triggered excessive autophagy in endothelial cells may contribute to the vascular pathology observed in PE. Therefore, modulating autophagy could be a potential therapeutic strategy to alleviate endothelial dysfunction in PE.

### 6.3 Interactions Between Oxidative Stress, Autophagy, and Apoptosis in PE

Excessive oxidative stress is a hallmark of PE, driven by an overproduction of reactive oxygen species (ROS) due to hypoxia, ischaemia/reperfusion, or mitochondrial dysfunction (Figure 2) [105]. In early PE, impaired trophoblastic invasion and defective spiral artery remodelling create a hypoxic placental microenvironmen [1, 2, 7, 9]. This hypoxia disrupts the mitochondrial electron transport chain, causing electron leakage and generating ROS, such as superoxide ions. These ROS inflict oxidative damage on DNA, proteins, and lipids, while activating signalling pathways that promote inflammation, endothelial dysfunction, and apoptosis, perpetuating a vicious cycle that exacerbates PE severity [105]. Studies have quantified this oxidative imbalance alongside reduced antioxidant enzyme activity, such as catalase. This oxidative stress environment sets the stage for altered cellular responses, including autophagy and apoptosis.

Autophagy serves as an essential mediator of oxidative stress by removing damaged components to maintain cellular homeostasis [19]. In response to excessive oxidative stress, cellular autophagy is heightened [14]. ROS can either oxidise or modify crucial autophagy‐regulatory proteins, such as AMP‐activated protein kinase (AMPK) and mammalian target of rapamycin (mTOR) complexes, thereby altering their activity and influencing the transcription and translation of genes associated with autophagy [106]. Specifically, ROS activate AMPK through phosphorylation, inhibiting mTOR, a negative regulator of autophagy, thereby promoting autophagy as a survival mechanism. Additionally, oxidative stress triggers the activation of transcription factors, such as nuclear factor erythroid 2‐related factor 2 (Nrf2), which, in turn, enhances the expression of antioxidant response element (ARE)‐targeted genes, including certain genes involved in autophagy [107].

Through mitophagy, autophagy selectively removes damaged mitochondria, reducing ROS production and stabilising intracellular ROS levels [19]. However, while short‐term autophagy activation serves as a protective mechanism against oxidative stress‐induced cellular damage, prolonged and excessive autophagy activation may result in detrimental consequences, such as resource depletion, functional impairment, exacerbated hypoxia, and lead to cell death [19]. For instance, hypoxia‐induced microRNA‐141‐3p promotes autophagy in trophoblast cells, contributing to poor placentation in preeclamptic patients [31, 108]. Similarly, excessive autophagy inhibits EVT, further compromising placental development [14].

Oxidative stress also drives apoptosis, shifting cellular responses from survival to programmed cell death. ROS directly damage mitochondrial DNA and membranes, triggering the release of cytochrome c and activating caspase cascades, which initiate intrinsic apoptosis [109, 110]. Additionally, oxidative stress induces endoplasmic reticulum (ER) stress, which can trigger apoptosis [111]. In PE, this shift is evident in increased pro‐apoptotic proteins (e.g., Bax, caspase‐3) and decreased anti‐apoptotic proteins (e.g., Bcl‐2) in trophoblasts [112].

The balance between autophagy and apoptosis is delicate. Excessive autophagy can lead to autophagic cell death, sharing morphological features with apoptosis [113]. Moreover, autophagy proteins like Beclin‐1 interact with apoptosis regulators such as Bcl‐2, influencing the switch between survival and death pathways [113]. In PE, this balance is disrupted, leading to inadequate trophoblast invasion and placental dysfunction.

Recent research found excessive BCL‐2‐related ovarian killer (BOK) in this interplay [114]. Overexpression of BOK under hypoxic and oxidative stress conditions disrupts the BOK‐Myeloid cell leukaemia‐1 (MCL1) balance, leading to ceramide overload and excessive trophoblast autophagy and apoptosis [115]. Similarly, hypoxia‐induced speckle‐type POZ protein (SPOP) impairs trophoblastic mobility via the PI3K/AKT pathway, contributing to placental pathology [116]. Meanwhile, a study has highlighted peroxiredoxin 1 (PRDX1) as a critical regulator [117]. PRDX1 expression is significantly reduced in placental trophoblasts from preeclamptic pregnancies compared to normotensive controls. In vitro experiments using HTR‐8/SVneo cells demonstrated that PRDX1 knockdown under oxidative stress (induced by H_2_O_2_) increases ROS levels, reduces autophagy (evidenced by decreased LC3II and Beclin1 expression), and enhances apoptosis (indicated by elevated cleaved‐Caspase3 and Bax expression) [118]. PRDX1 modulates these effects through the PTEN/AKT signalling pathway, where its knockdown decreases PTEN and increases phosphorylated AKT, linking oxidative stress to altered autophagy and apoptosis [119]. These findings suggest that PRDX1 plays a protective role by maintaining autophagy and mitigating apoptosis, and its deficiency may exacerbate PE pathogenesis.

The dynamic interplay among oxidative stress, autophagy, and apoptosis is central to PE's pathogenesis. Autophagy initially serves as a protective mechanism against oxidative damage, but sustained stress can tip the balance toward apoptosis, leading to trophoblast dysfunction and clinical manifestations of PE. The identification of PRDX1 as a regulator opens new avenues for therapeutic exploration, potentially targeting the PTEN/AKT pathway to restore cellular balance.

### 6.4 ER Stress in PE

Endoplasmic reticulum (ER) stress occurs due to an imbalance between the unfolded protein load and ER folding capacity, leading to the activation of the unfolded protein response (UPR) to restore ER homeostasis (Figure 2) [120]. Autophagy is also induced by the unfolded protein response and ER stress [9, 120]. The major transducers of UPR that regulate autophagy are protein kinase R‐like ER kinase (PERK), activating transcription factor 6 (ATF6), and inositol‐requiring transmembrane kinase/endonuclease 1 (IRE1α) [120]. Moderate ER stress is essential for placental development, while low or excessive ER stress can affect placental growth [9].

Accumulating evidence has emerged suggesting that dysregulated ER stress and UPR activity are associated with PE. In preeclamptic placentas, ER cisternae are significantly dilated, and the presence of amorphous proteinaceous precipitates indicates signs of ER stress [121]. Excessive ER stress inhibits autophagy. For example, Nakashima et al. showed that excessive ER stress decreased lysosomal numbers in trophoblast cell lines, resulting in the accumulation of autophagosomes and a decrease in autolysosomes, which is a sign of suppressed autophagic flux [122]. Additionally, excessive ER stress leads to the overexpression of PERK and IRE1α, activating more transcription factors ATF4 and ATF6, which decreases the secretion of placental growth factor (PlGF) in preeclamptic placentas [123]. Decreases in PlGF mRNA were also observed in placenta‐specific Atg7 knockout mice [67]. On the other hand, autophagy inhibition exacerbates ER stress in trophoblast cells [15, 52]. The ER may also play a role in autophagy through the dysregulation of sphingolipids. Synthesis of sphingolipids in the endoplasmic reticulum and ceramide overload accompanied by increased trophoblast cell autophagy have been found in PE [35]. Furthermore, ER stress induces protein aggregates, which can be eliminated by the lysosome‐autophagy system and ER‐phagy [124].

### 6.5 Protein Aggregation and Cellular Senescence in PE

Recent studies have identified protein aggregation in PE, with proteomic analyses revealing numerous abnormally aggregated proteins in affected placentas (Figure 2) [125]. These aggregates infiltrate trophoblasts, causing cytotoxicity and compromising normal trophoblast function. Notably, proteins typically associated with neurodegeneration, such as transthyretin (TTR), amyloid β, and hyperphosphorylated tau proteins, have been detected in the serum and urine of preeclamptic patients [126]. Excessive ER stress, hyperactive UPR, and impaired autophagy‐lysosomal machinery contribute to TTR aggregate accumulation, as demonstrated by Cheng et al. [52]. Transgenic mice overexpressing wild‐type human TTR exhibit placental TTR aggregate deposition and PE‐like symptoms [52]. Autophagy‐deficient cells exhibit increased protein aggregate deposition, leading to PE‐like manifestations in IL‐10 knockout mice. Depletion of serum protein aggregates mitigates cytotoxicity [127]. Autophagy plays a vital role in maintaining cellular protein quality and placental homeostasis by preventing protein aggregation [21]. Misfolded or aggregated proteins are targeted for clearance via the ubiquitin‐proteasome system and autophagy‐lysosome system [21]. Impaired autophagy‐lysosomal function and reduced p97/VCP contribute significantly to aggregate accumulation [53]. TFEB‐mediated lysosomal biogenesis is compromised in preeclamptic placentas, accompanied by increased aggregate deposition, as confirmed by Nakashima et al. [55]. Placenta‐specific Atg7 knockout mice and autophagy‐deficient trophoblasts display TFEB downregulation and enhanced aggregate deposition [54, 67]. p97/VCP, involved in autolysosome‐lysosome fusion, is decreased in preeclamptic placentas, concurrent with ubiquitin protein accumulation [53]. Autophagy dysregulation exacerbates trophoblast dysfunction under hypoxic conditions [54, 78].

Protein aggregates may disrupt placental development by inducing cellular senescence. Increased placental senescence, marked by telomere attrition and elevated senescence‐associated β‐galactosidase activity, is a hallmark of PE, as proposed by Cox and Redman [128]. Aggregates trigger senescence via oxidative stress and DNA damage, activation of the p53‐p21 pathway, disruption of proteostasis, and induction of the SASP, characterised by pro‐inflammatory cytokine and growth factor secretion [129]. The SASP contributes to endothelial dysfunction, inflammation, and other PE‐related pathologies [129].

Autophagy, oxidative stress, ER stress, protein aggregation and cellular senescence are interconnected processes that play important roles in the pathogenesis of PE. Dysregulation of these processes in the placenta can lead to placental dysfunction, oxidative stress, inflammation, endothelial dysfunction, and other hallmarks of PE. Understanding the molecular mechanisms underlying these processes may provide new insights into the development of therapeutic strategies for PE. Further research is needed to elucidate the specific mechanisms involved and to identify potential targets for intervention.

## 7 Autophagy in Different Immune Cells

The immune system at the maternal‐fetal interface is pivotal for sustaining pregnancy and ensuring immune tolerance toward the semi‐allogeneic fetus. Among the diverse immune cell populations, decidual natural killer (dNK) cells, macrophages, and regulatory T (Treg) cells are particularly prominent due to their abundance and critical functions [130]. The dNK cells, constituting 50%–70% of decidual leukocytes, drive spiral artery remodelling and trophoblast invasion. Macrophages, comprising 20%–30% of leukocytes, support tissue remodelling and immune tolerance through their M1/M2 polarisation states. Treg cells suppress excessive immune responses, safeguarding the fetus from maternal immune rejection [130]. Dysregulation of these cells is closely linked to pregnancy complications such as PE. Thus, exploring how autophagy—a conserved process maintaining cellular homeostasis—regulates their functions is essential for understanding PE pathogenesis and identifying therapeutic targets.

### 7.1 Autophagy and NK Cells

**FIGURE 4:**
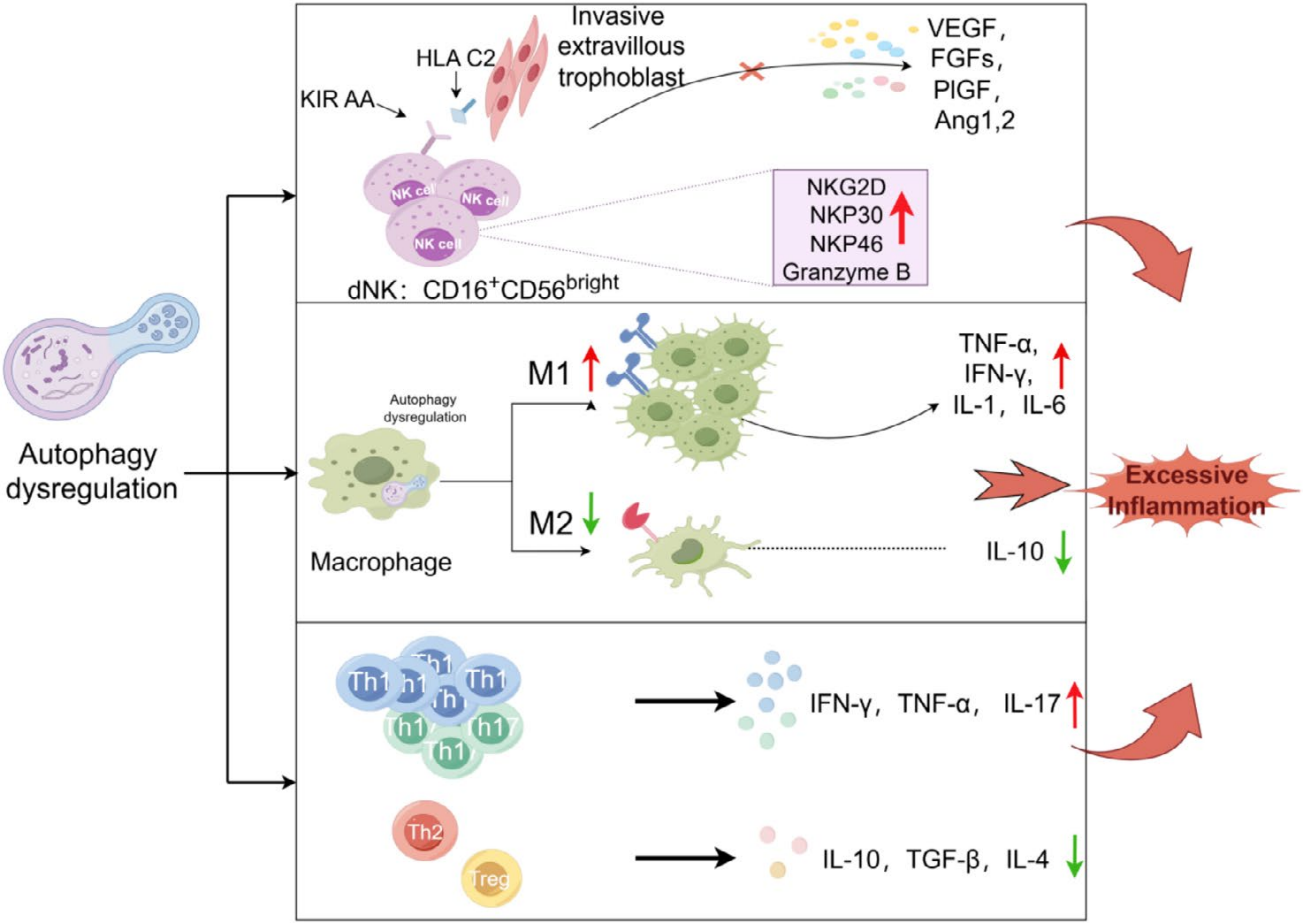
Immune changes regulated by autophagy. Dysregulation of autophagy in the PE leads to immune cell dysfunction. NK cells: Inhibit the production of angiogenesis factors and increase surface expression of natural killer receptors, which enhancing their lytic activity. Macrophages: Activation of pro‐inflammatory M1 macrophages occurs. T cells: Proinflammatory TH1 and TH17 cells increase, which secreting excessive TNF‐α, IFN‐γ, and IL‐17. These responses all exacerbate systemic inflammatory immune responses and tissue damage in the placenta, while also inhibiting placental vascular development, ultimately leading to PE.

The dNK cells, representing most of the immune cells at the fetal‐maternal interface during early pregnancy, constitute the most abundant cell type in the human decidua (Figure 4) [130]. Unlike peripheral blood NK cells, which are predominantly CD16^+^CD56^dim^, dNK cells are mainly CD56^bright^CD16^−^, exhibiting reduced cytotoxicity and secreting angiogenic factors like VEGF, FGFs, PIGF, and Ang‐1/2 to promote spiral artery remodelling and EVT invasion [131]. The proportion of CD56 bright dNK cells dynamically shifts during pregnancy, influenced by hypoxia and cytokine profiles in the decidual microenvironment. Conversely, CD56 dim NK cells, though less prevalent, may increase in pathological conditions, displaying heightened cytotoxic potential [130]. Studies show that when maternal NK cells expressing the KIR AA genotype and interacting with trophoblasts expressing HLA‐C2 have been associated with an increased risk of PE, potentially due to excessive inhibition of dNK cells, reduced production of angiogenic factors and cytokines, and consequently, impaired placentation and arterial transformation [131]. Animal models confirm that dNK cell depletion or impaired proliferation leads to defective vascular remodelling [131].

Autophagy subtly modulates dNK‐trophoblast interactions [11]. Autophagy‐deficient trophoblasts upregulate IGF‐2, enhancing NK cell‐activating receptors (e.g., CD16, NKG2D, NKp30, NKp46), increasing cytotoxicity, and potentially causing pregnancy loss [132]. This process involves the IGF2‐TP53‐CXCR4 axis, where autophagy inhibition reduces CXCR4+ dNK cells, critical for immune tolerance [133]. NKG2D, a key NK cell activating receptor, binds to its ligand ULBP1, skewing the balance of cytokines (TNF‐α, TGF‐β1, IFN‐γ, IL‐8, and IL‐6) secreted by NK cells in PE, potentially suppressing EVT invasion [134]. Although direct evidence of autophagy within dNK cells is limited, studies on peripheral NK cells indicate that autophagy is essential for differentiation and activation. For instance, Atg5 deficiency causes mitochondrial damage and excessive ROS, impairing NK cell development [135]. In the hypoxic decidual environment, autophagy likely supports CD56bright dNK cell function, maintaining their immune‐tolerant phenotype. Additionally, autophagy in decidual stromal cells during decidualisation enhances dNK cell residence and enrichment, underscoring the decidual microenvironment's role in NK cell modulation [136]. Future research should explore whether autophagy directly regulates dNK cell differentiation or activation to clarify its contribution to maternal‐fetal immune tolerance.

### 7.2 Autophagy and Macrophages

Macrophages account for 20%–30% of decidual leukocytes, ranking second to NK cells [130]. Their principal functions include secreting cytokines, growth factors, proteases, angiogenesis, and tissue remodelling factors, contributing VEGF, MMPs, and angiogenins to support fetal blood supply establishment [137]. Decidual macrophages orchestrate maternal‐fetal immune tolerance and promote spiral artery remodelling by modulating immune responses and supporting vascular restructuring [138]. Autophagy sustains immune homeostasis and macrophage functions, such as pattern recognition, cytokine release, inflammasome activation, and LC3‐associated phagocytosis [139]. Disrupted autophagy in macrophages disrupts these regulatory processes, triggering excessive inflammation and contributing to pathological conditions [140].

In PE patients, the numbers of macrophage subtypes were changed, and the polarisation states of the macrophages were different compared to normal pregnancy patients (Figure 4) [141]. This M1/M2 imbalance results from autophagy dysregulation. Autophagy promotes M2 polarisation via AMPK/mTOR and STAT6 pathways, reducing mitochondrial ROS through USP19‐mediated autophagy flux, favouring M2‐like phenotypes [141, 142, 143]. In PE, reduced M2 macrophages correlate with upregulated autophagy‐related genes (e.g., PKM, LEP, HK2), suggesting impaired autophagy contributes to M1/M2 imbalance. Autophagy also suppresses pro‐inflammatory M1 responses by regulating NF‐κB signalling through p62‐dependent mechanisms [141].

The lysophosphatidic acid (LPA)‐autophagy axis is critical for decidual macrophage function. ENPP2‐LPA activation via LPAR1/PPARG‐DDIT4‐autophagy axis upregulates adhesion factors (e.g., cadherins, selectins) in a CLDN7‐dependent manner, promoting macrophage adhesion, retention, and M2 differentiation during normal pregnancy. Defects in this axis increase miscarriage risk by restricting macrophage residence [144]. In recurrent spontaneous abortion, Grim‐19 deficiency enhances autophagy in decidual macrophages, upregulating Beclin1, LC3B II/I, and BNIP3, correlating with pro‐inflammatory cytokine production [145]. While pregnancy‐specific data on autophagy‐related non‐coding RNAs are limited, studies in other contexts suggest miRNAs (e.g., miR‐205‐5p) and lncRNAs modulate autophagy and macrophage polarisation. For instance, miR‐205‐5p promotes autophagy in alveolar macrophages, inhibiting fibrosis, suggesting a potential role in decidual macrophages [146]. Future studies should investigate whether non‐coding RNAs regulate autophagy‐driven macrophage polarisation in pregnancy.

### 7.3 Autophagy and T Cells

T lymphocytes, comprising 10%–20% of decidual lymphocytes, are recruited to regulate maternal‐fetal immunity during pregnancy (Figure 4) [130]. CD4+ T helper cells and CD8+ cytotoxic T cells make up 35%–45% and 45%–75% of these cells, respectively [147]. Effector CD4+ T helper cells include Th1, Th2, Th17, and Treg subsets, each with unique cytokine profiles and functions [130, 147].

Healthy pregnancies promote Th2 and Treg cell differentiation via progesterone production from the placenta, while PE skews this differentiation toward pro‐inflammatory Th1 and Th17 phenotypes [147]. A Th1/Th2 and Th17/Treg imbalance, shifting toward a Th1/17‐dominated state, leads to the production of cytokines like IL‐2, IFN‐γ, TNF‐α, and IL‐17, which activate CD8+ T cells and NK cells, enhancing cytotoxic activity [148]. Autophagy acts as an effector in Th1/Th2 polarisation, with Th1 cytokines (IFN‐γ, TNF‐α) inducing autophagy, while Th2 cytokines (IL‐4, IL‐10) inhibit it [149]. Autophagy‐deficient CD4+ T cells lose the capacity to differentiate into Th1 cells, attributed to reduced mitochondrial activity due to dysfunctional autophagy and decreased IFN‐γ production [150]. Impaired mitochondrial autophagy in CD4+ T lymphocytes disrupts redox metabolism and elevates Th17 cytokines, leading to inflammation [150].

Tregs, identified by CD4, CD25, and FOXP3 expression, are central to immune tolerance during pregnancy [130]. In healthy pregnancies, Treg numbers increase to suppress anti‐fetal immune responses, while in PE, Treg cells decrease, with elevated pro‐inflammatory Th1 and Th17 responses [148]. Autophagy enhances Treg differentiation and function primarily through the AMPK/mTOR pathway. mTOR inhibition, facilitated by autophagy activation, upregulates FOXP3 expression and Treg suppressive function [149]. Mechanistically, autophagy stabilises FOXP3 by modulating oxidative stress and metabolic pathways, such as AMPK/mTOR signalling [150, 151]. In PE, AMPK/mTOR pathway dysregulation impairs Treg differentiation, contributing to immune imbalance. Elevated sEng in PE inhibits autophagy, disrupting Treg differentiation [152]. Furthermore, autophagy supports Treg function through epigenetic modifications, including CpG demethylation and histone modifications in Treg‐specific super‐enhancer regions, ensuring stable FOXP3 expression [153]. Thus, autophagy is critical for Treg differentiation and function at the maternal‐fetal interface, supporting immune tolerance.

In conclusion, autophagy plays a multifaceted role in coordinating immune cell functions at the maternal–fetal interface. In trophoblast cells, autophagy regulates IGF‐2 secretion to limit dNK cell cytotoxicity, promoting immune tolerance [132]. In macrophages, autophagy drives M2 polarisation through STAT6 and NF‐κB pathways, supporting anti‐inflammatory responses and tissue remodelling [141]. In T cells, autophagy enhances Treg differentiation via the AMPK/mTOR pathway, stabilising FOXP3 expression [150]. Additionally, autophagy may modulate dendritic cell function through MHC‐II antigen presentation. For instance, autophagy delivers intracellular proteins to lysosomes for degradation, providing peptides for MHC‐II presentation by dendritic cells, which is critical for presenting self and pathogen‐derived antigens to CD4+ T cells [154]. Dysregulation of autophagy in these cell types may disrupt the delicate balance of immune tolerance, contributing to pregnancy complications such as PE [155]. Future research should focus on elucidating the specific mechanisms by which autophagy regulates immune cell crosstalk at the maternal–fetal interface and explore whether targeting autophagy can offer therapeutic benefits for pregnancy‐related disorders.

## 8 Autophagy and Maternal Factors

Revised two‐stage model of PE accommodates most known risk factors including advanced maternal age, chronic pre‐pregnancy disease (e.g., obesity), and other pregnancy risk factors (including multiples) [156]. Maternal pre‐conceptional health status, including age, body mass index (BMI), and multiples pregnancies, appears to be associated with dysregulated autophagy, which in turn increases the risk of PE. Specifically, studies found that expression of key autophagy‐related hub genes (PKM, LEP) was significantly higher in women over 35 years old with PE compared to those under 35 [16]. Additionally, studies have revealed that increased BMI and obesity, which are strongly associated with PE, can impact placental autophagy, accompanied by sex‐specific effects [157]. Two lines of evidence support the view. First, reduced placental autophagy appears to be observed in obese women compared to normal weight controls [158]. Second, placentas from male foetuses of obese mothers show increased autophagy activation and autophagy impairment was more pronounced in placentas of male foetuses compared to females [157]. In addition, impaired autophagy in EVT and impaired vascular remodelling of uterine spiral arteries were observed in oocyte donation pregnancies, regardless of the presence or absence of PE [159]. Multiple gestations complicated by twin‐to‐twin transfusion syndrome or selective fetal growth restriction are at a greater risk for PE [160]. Mao et al. demonstrate that twin anaemia‐polycythemia sequence (TAPS) placentas display intertwin autophagy discordance, suggestive of inhibition of autophagic activity in the territory of the anaemic twin with associated accumulation of lysosomes and autolysosomes [161].

Genetic factors also significantly contribute to the dysregulation of autophagy for PE. For example, upregulation of METTL14 in preeclamptic placentas inhibits trophoblast invasion and enhances autophagy in HTR‐8/SVneo cells by promoting m(6)A methylation of target genes, thereby disrupting placental development [162]. Importantly, these epigenetic alterations can be triggered by various environmental factors. Maternal cadmium exposure was found to activate placental mitophagy and was associated with the occurrence of PE [163]. Additionally, maternal titanium dioxide nanoparticles exposure triggered PE‐like symptoms, potentially by promoting excessive autophagy and hindering invasion in trophoblast cells [164]. Moreover, studies have demonstrated that folate deficiency can induce abnormal placentation and autophagy plays a key role in the process [165].

In addition to maternal factors, fetal factors significantly contribute to the regulation of placental autophagy and the risk of PE. Given that the placenta is of fetal origin, genetic and epigenetic alterations within the placenta can be regarded as fetal factors. Research has demonstrated that deficiencies in autophagy‐related genes, such as Atg7, in the placenta lead to impaired autophagy, resulting in PE‐like symptoms in animal models. Specifically, placenta‐specific Atg7 knockout mice display hypertension and reduced placental growth factor levels, mirroring human PE characteristics (Trophoblast‐Specific Atg7 Knockout) [67]. Furthermore, conditions like fetal growth restriction, often associated with PE, have been correlated with increased placental autophagy. This suggests that fetal stress or genetic predispositions can trigger dysregulated autophagy, thereby contributing to PE pathogenesis. Therefore, both maternal and fetal factors interact through the modulation of placental autophagy to influence the development of PE.

## 9 Targeting Autophagy for PE Treatment

While treatment options for PE are limited, ongoing research is exploring novel therapies that target autophagy. Some potential approaches include the use of Rapamycin, Chloroquine, ULK1 Inhibitors, aspirin, statins, metformin, proton pump inhibitors, and natural small molecules (Table 2) [191].

**TABLE 2:** Potential treating methods for PE targeting autophagy.

Drug/agent	Model	Potential effects	Targeted pathway/mechanism
ANP [32]	HTR‐8/SVneo, JEG3 cells	Inhibits autophagy via AMPK/mTORC1, enhances invasion	AMPK/mTOR (enhances iEVT invasion)
Amlexanox [166]	LPS‐induced mice, HTR8/SVneo, Sw.71 cells	Regulates TBK1‐mTORC1, reduces placental inflammation	TBK1/mTORC1 (reduces inflammation)
Bioflavonoid luteolin [167]	PE placental explants	Reduces sFlt‐1 via PI3K/Akt/HIF‐1α inhibition	PI3K/Akt/HIF‐1α (enhances enEVT vascular remodelling)
Chloroquine [168]	H_2_O_2_‐treated BeWo cells	Sustains p62‐NBR1‐Nrf2 autophagy, reduces oxidative stress	Nrf2 pathway (mitigates oxidative stress)
Cyclosporin A [169]	L‐NAME‐induced mice, H/R‐treated HTR8/SVneo cells	Upregulates autophagy, reduces apoptosis, senescence	Autophagy activation (enhances trophoblast survival)
Esomeprazole [170]	L‐NAME‐induced mice, hypoxia‐treated HTR8 cells	Inhibits AMPKα‐mTOR‐dependent autophagy, improves endothelial function	AMPK/mTOR (enhances enEVT vascular remodelling)
Irisin [171]	H/R‐treated JEG3 cells	Activates Akt, reduces oxidative stress, apoptosis	Akt signalling (reduces oxidative stress)
Ligustrazine [172]	LPS‐induced rats, JEG3 cells	Inhibits autophagy, promotes trophoblast viability, migration	Autophagy modulation (enhances iEVT invasion)
Melatonin [173]	H/R‐treated primary villous trophoblasts	Activates autophagy, increases syncytiotrophoblast survival	Autophagy activation (enhances trophoblast survival)
Metformin [174]	LPS‐induced HTR8/SVneo cells	Triggers autophagy, reduces inflammation, oxidative stress	AMPK activation (enhances iEVT invasion, reduces inflammation)
MSCs [173]	Hypoxic JAR, JEG‐3, HTR‐8 cells	Activates JAK2/STAT3, promotes trophoblast survival	JAK2/STAT3 (enhances iEVT survival)
MSCs [175]	Hypoxic JEG‐3, HTR‐8 cells	Inactivates EZH2‐dependent mTOR, promotes trophoblast survival	mTOR inhibition (enhances iEVT survival)
NSAIDs [176]	L‐NAME‐induced rats	Activates PI3K/Nrf2/HO‐1, reduces blood pressure, proteinuria	Nrf2/HO‐1 (reduces inflammation, oxidative stress)
Peptide‐PDCC4 [177]	LPS‐induced rats, TNFα‐treated HUVECs	Activates PI3K/mTOR/HIF‐1α, relieves endothelial dysfunction	PI3K/mTOR/HIF‐1α (enhances enEVT vascular remodelling)
Progesterone [178]	L‐NAME‐induced rats, PE serum‐treated HTR8/SVneo cells	Activates PI3K/Akt, reduces inflammation	PI3K/Akt (enhances iEVT invasion, reduces inflammation)
Progesterone [169]	SIRT1‐knockdown mice, HTR‐8/SVneo	Promotes invasion, inhibits apoptosis, alleviates PE symptoms	SIRT1/PI3K/Akt (enhances iEVT invasion)
Quercetin [179]	LPS‐induced rats	Reduced blood pressure, proteinuria, and alleviate oxidative stress and inflammation	(reduces oxidative stress and inflammation)
Rapamycin [180]	LPS‐induced mice, and L‐NAME‐induced mice	Induces autophagy, reduced blood pressure, proteinuria and improved blood lipid	mTOR inhibitor (improves maternal factors)
Resveratrol [181]	H_2_O_2_‐treated HTR8/SVneo cells	Activates autophagy, reduces oxidative stress, apoptosis	Autophagy activation (enhances iEVT survival)
Resveratrol [182]	H_2_O_2_‐treated HTR8/SVneo cells	Decreases autophagy, mitigates oxidative stress	Autophagy modulation (reduces oxidative stress)
Salvianolic acid B [183]	H_2_O_2_‐treated HTR8/SVneo cells	Activates PI3K/Akt, promotes invasion, migration	PI3K/Akt (enhances iEVT invasion)
Salvianolic acid B [184]	Endotoxin‐induced rats, H/R‐treated HTR‐8/SVneo cells	Activates CXCR4/Akt, improves blood pressure, enhances invasion	CXCR4/Akt (enhances iEVT invasion, enEVT remodelling)
Trehalose [185]	PE serum‐induced IL‐10−/− mice, hypoxia‐exposed primary trophoblasts	Normalises autophagy, inhibits protein aggregation, increases TFEB	TFEB‐mediated autophagy (enhances iEVT invasion, enEVT remodelling)
Vitamin D [186]	H/R‐treated HTR‐8/SVneo cells	Upregulates LAMP3, promotes invasion, inhibits apoptosis	Autophagy‐lysosome pathway (enhances iEVT invasion)
Vitamin D [187]	H/R‐treated HTR‐8/SVneo cells	Activates autophagy, inhibits pyroptosis, enhances invasion, angiogenesis	Autophagy activation (supports iEVT invasion, enEVT remodelling)
Vitamin D [188]	RUPP‐induced rats	Increases autophagy, reduces apoptosis, improves placental ischemia	Autophagy activation (enhances placental homeostasis)
3‐MA [189]	RUPP‐induced rats	Inhibits autophagy, reduces blood pressure, proteinuria	Autophagy inhibition (improves maternal factors)
6‐Gingerol [190]	L‐NAME‐induced mice, H/R‐treated HTR‐8/SVneo cells	Suppresses BNIP3‐dependent mitophagy, reduces apoptosis	BNIP3‐mediated mitophagy (reduces oxidative stress)

Abbreviations: 3‐MA, 3‐Methyladenine; Akt, protein kinase B, also known as AKT; AMPK, adenosine 5′ monophosphate‐activated protein kinase; ANP, atrial natriuretic peptide; BNIP3, BCL2/adenovirus E1B19kDa protein‐interacting protein 3; CXCR4, C‐X‐C motif chemokine receptor 4; enEVT, endovascular extravillous trophoblast cell; EZH2, Enhancer of zeste homologue 2; H/R, hypoxia/reoxygenation; H_2_O_2_, Hydrogen peroxide; HIF‐1α, Hypoxia‐inducible factor 1 α; HO‐1, Heme Oxygenase‐1; HUVEC, Human Umbilical Vein Endothelial Cells; iEVT, interstitial extravillous trophoblast cell; IL, Interleukin; JAK2, janus kinase 2; LAMP, lysosomal‐associated membrane protein; L‐NAME, NG‐Nitro‐L‐arginine Methyl Ester, Hydrochloride; LPS, Lipopolysaccharide; MSCs, mesenchymal stem cells; mTOR, mammalian target of rapamycin; mTORC1, mTOR complex 1; Nrf2, nuclear factor‐erythroid 2‐related factor 2; NSAIDs, Nonsteroidal anti‐inflammatory drugs; PDCC4, peptide derived from complement C4 A chain; PE, PE; PI3K, Class III phosphatidylinositol 3‐kinase complex; RUPP, reduced uterine perfusion pressure; sFlt‐1, Soluble fms‐like tyrosine kinase‐1; SIRT1, silent information regulator sirtuin 1; STAT3, signal transducer and activator of transcription; TBK1, TANK‐binding kinase 1; TFEB, Transcription factor EB; TNFα, Tumour Necrosis Factor‐α.

### 9.1 Autophagy‐Modulating Drugs in PE Research

Rapamycin, an mTOR inhibitor that induces autophagy, has been investigated in PE‐like mouse models. In a study by Yi et al., rapamycin was administered to mice and significantly reduced 24‐h urinary protein levels and improved blood lipid profiles (e.g., reduced free fatty acids and triglycerides) in the preimplantation and early pregnancy groups [180]. However, no specific clinical trials of rapamycin for PE in humans have been reported. Case reports indicate that rapamycin is safe during pregnancy in organ transplant patients, but its efficacy for PE remains to be tested.

Chloroquine, a classical autophagy inhibitor, has been shown to restore endothelial function and reduce oxidative stress by preserving the NBR1‐p62‐Nrf2 autophagy pathway [192]. Its derivative, hydroxychloroquine, is commonly used to treat systemic lupus erythematosus (SLE) during pregnancy. Observational studies suggest that hydroxychloroquine may reduce the risk of PE in women with SLE. Seo et al. reported that hydroxychloroquine treatment during pregnancy was associated with an 89% reduction in the risk of PE in SLE patients compared to those not treated [193]. Additionally, Sciascia et al. and Mekinian et al. have shown reduced placenta‐mediated complications and lower rates of PE/HELLP syndrome with hydroxychloroquine in patients with antiphospholipid antibodies [194]. Ongoing clinical trials, such as the HYPATIA trial, aim to evaluate hydroxychloroquine's role in preventing PE in high‐risk pregnancies. However, its efficacy in the general population remains under investigation, and further research is needed to confirm these findings [195].

ULK1 (Unc‐51 like autophagy activating kinase 1) is a key initiator of autophagy. While ULK1 inhibitors have been developed and studied in diseases such as cancer and chronic myeloid leukaemia, there are currently no reports of their use in PE models or clinical trials [196, 197]. Given the role of autophagy in PE pathogenesis, ULK1 inhibitors represent a potential area for future research.

### 9.2 Other Autophagy‐Regulating Agents

Metformin, an anti‐diabetic drug, has shown promise in reducing sFlt‐1 and sEng levels, improving placental perfusion, and rebalancing angiogenic and antiangiogenic factors [191, 198]. It also activates AMPK phosphorylation to mitigate inflammatory injury and oxidative stress by inhibiting NF‐κB/sFlt‐1 and Nrf2/HO‐1 signalling pathways in LPS‐stimulated HTR‐8/SVneo cells [199].

Esomeprazole, a proton pump inhibitor considered safe during pregnancy, has demonstrated in preclinical studies the ability to downregulate sFlt‐1 and sEng levels [200, 201]. It also inhibits the SIRT1/AMPKα‐mTOR autophagy pathway, thereby reducing endothelial dysfunction and promoting vasodilation [170].

Aspirin is currently the only medication recommended for PE prevention [3]. Combining apocynin and aspirin therapy has been shown to activate the PI3K/Nrf2/HO‐1 signalling pathway, alleviating hypertension and proteinuria in pregnant rats with PE symptoms [176]. Nonsteroidal anti‐inflammatory drugs (NSAIDs) can alleviate cardiac and renal damage by regulating autophagy signalling pathways, mitigating inflammation, and reducing oxidative stress [176].

Resveratrol, a naturally occurring phytoalexin found in grapes, nuts, and red wine, can induce autophagy through SIRT1 activation [182]. Wang P et al. demonstrated that resveratrol reduces H_2_O_2_‐induced oxidative stress and apoptosis through SIRT1‐dependent autophagy [181].

Vitamin D deficiency has been noted in PE patients, and there is a correlation between deficiency and reduced survival capacity [187]. Tian X et al. demonstrated that vitamin D promotes trophoblast cell viability, invasion, and inhibits apoptosis by upregulating LAMP3 expression [186]. It also ameliorates placental ischaemia and organ damage [188].

Another potential approach is the use of mesenchymal stem cells (MSCs), which have shown promise as a cell‐based therapy [202]. MSCs can activate JAK2/STAT3 signalling and deactivate EZH2‐dependent mTOR signalling, stimulating trophoblast proliferation and invasiveness [173, 175]. However, further research is needed to explore the effectiveness and safety of this approach.

Furthermore, natural small molecules such as quercetin and trehalose have shown potential in preclinical studies. Quercetin, a bioflavonoid found in various fruits and vegetables, possesses anti‐inflammatory and antioxidant properties. In a LPS‐induced PE rat model, quercetin treatment significantly reduced systolic blood pressure, proteinuria, and pro‐inflammatory cytokine production [179]. Additionally, quercetin enhanced the anti‐inflammatory effects of aspirin in this model, suggesting a synergistic effect. Although the direct role of quercetin in regulating autophagy in PE has not been fully validated, its ability to alleviate oxidative stress and inflammation, which are closely linked to autophagy dysregulation, makes it a candidate for further research [203]. Therefore, quercetin is a natural compound with therapeutic potential for PE, warranting further investigation into its mechanisms, including its effects on autophagy.

Trehalose, a natural disaccharide, has been shown to correct autophagy by activating transcription factor EB (TFEB), a key regulator of autophagy and lysosomal biogenesis [185, 204]. In preclinical studies using both in vitro and in vivo models of PE, trehalose normalised blood pressure, reduced proteinuria, improved fetal growth, and decreased sFlt‐1 levels. It also inhibited protein aggregation and restored lysosomal markers [205]. Furthermore, RNA‐seq analysis revealed that trehalose rescued the expression of several genes dysregulated in PE, including those involved in RhoA, FAK, and fibrosis signalling pathways [205].

In addition to the above drugs, there are some hormone substances including progesterone, ANP, and melatonin that can regulate autophagy, suggesting a potential role for treatment for PE [32, 169, 174, 178, 206, 207, 208, 209]. Moreover, Chinese medicine such as ligustrazine, 6‐Gingerol, and Salvianolic acid B were thought to be a promising therapy for PE [167, 171, 172, 183, 184, 190]. Data from animal models of PE demonstrated that Chinese medicine compounds alleviated trophoblast invasion ability [171, 183]. Some immune regulators, such as cyclosporin A, amlexanox, new peptides, and some inhibitors, have potential prompts including reversal of angiogenic imbalance, improvement of endothelial function, or prevention of injury from oxidation and inflammation [166, 168, 177, 189]. These medicines may act as adjunctive therapies to reduce the risk of PE. However, their research seems to be more cautious among pregnant women.

### 9.3 Therapeutic Timing and Stage‐Specific Applications

The timing of intervention is critical in the management of PE. Preventive strategies are generally more effective when initiated early in pregnancy [210]. Low‐dose aspirin is recommended starting from 12 weeks of gestation for high‐risk women [211, 212]. In animal models, rapamycin showed beneficial effects when administered during preimplantation and early pregnancy [180], suggesting that early intervention may be key for autophagy‐modulating therapies. Hydroxychloroquine, often used throughout pregnancy in SLE patients [193], may provide continuous protection against PE. However, for therapeutic interventions in established PE, the efficacy of autophagy‐modulating drugs requires further evaluation. The heterogeneity of PE, including early‐onset and late‐onset forms, suggests that stage‐specific applications may be necessary [213]. Future research should investigate the optimal timing and stage‐specific applications of these therapies to maximise their therapeutic benefits.

Autophagy‐modulating drugs offer a promising avenue for the treatment of PE. However, clinical evidence remains limited; large‐scale clinical trials are needed to confirm their safety and effectiveness. Determining the optimal timing and stage‐specific applications of these therapies will be essential for their successful translation into clinical practice. Future studies on more safe and effective research on target drugs in PE are warranted in PE treatment.

## 10 Conclusions

PE is a pregnancy‐specific disorder typically characterised by hypertension and proteinuria, which can lead to severe maternal and fetal complications [61]. Proper placental development and function are critical for a successful pregnancy, and disruptions in these processes contribute to the onset of PE. In PE, impaired trophoblast invasion and insufficient spiral artery remodelling lead to inadequate placental perfusion and placental ischemia/hypoxia [8, 214, 215]. The placental ischemia and hypoxia then trigger a cascade of complex cellular and molecular responses, including oxidative stress, ER stress, and ischemia‐reoxygenation injury et al., contributing to excessive systemic inflammatory response, triggering endothelial activation, intravascular inflammation, and the development of hypertension and other maternal symptoms associated with PE [7, 216, 217]. Thus, the progression from placental ischemia and hypoxia to oxidative stress and other events represents a critical pathway in the pathogenesis of PE [61, 215].

Within but not limited to this pathological environment of PE, autophagy initially plays a protective role by mitigating the effects of stress [12, 51, 214]. However, with sustained activation of autophagy, its role may shift from being protective to a disease‐promoting mechanism [14, 91]. Dysregulated autophagy can adversely affect trophoblast invasion, vascular remodelling, inflammation, immune response, and maternal factors, thereby exacerbating the pathological course of PE [12, 21, 54, 101]. In this context, aberrant autophagy ceases to be merely a protective mechanism and may instead become a driving force in the progression of the disease.

Through clarifying the role of autophagy in the pathogenesis of PE, this provides a direction for further understanding the underlying mechanisms of PE and identifying potential therapeutic targets, contributing to novel therapeutic strategies such as hormonal therapy or traditional medicine treatments in the future [32, 167, 169, 171, 172, 174, 178, 179, 183, 184, 190, 203, 206, 207, 208, 209].

## Author Contributions

M.X.: Writing – original draft preparation; Q.W., F.W., and H.L.: Writing – original draft edition; Z.L., X.H., and Z.H.: creating the original drawings and tables; L.K., H.M., Q.W., S.W., H.L., and M.L.: writing – review and editing; H.Y. and S.W.: supervision and final revision. All authors have read and agreed to the published version of the manuscript.

## Conflicts of Interest

The authors declare no conflicts of interest.

## Data Availability

Data sharing not applicable to this article as no datasets were generated or analysed during the current study.
